# Immunogenicity and protective efficacy of a rhesus adenoviral vaccine targeting conserved COVID-19 replication transcription complex

**DOI:** 10.1038/s41541-022-00553-2

**Published:** 2022-10-27

**Authors:** Gabriel Dagotto, John D. Ventura, David R. Martinez, Tochi Anioke, Benjamin S. Chung, Mazuba Siamatu, Julia Barrett, Jessica Miller, Alexandra Schäfer, Jingyou Yu, Lisa H. Tostanoski, Kshitij Wagh, Ralph S. Baric, Bette Korber, Dan H. Barouch

**Affiliations:** 1 Center for Virology and Vaccine Research, Beth Israel Deaconess Medical Center, Harvard Medical School, Boston, MA USA; 2 Harvard Medical School, Boston, MA USA; 3 Theoretical Biology and Biophysics, Los Alamos National Laboratory, Los Alamos, NM USA; 4 Department of Epidemiology, University of North Carolina at Chapel Hill, Chapel Hill, NC USA; 5 The New Mexico Consortium, Los Alamos National Laboratory, Los Alamos, NM USA; 6 Ragon Institute of MGH, MIT and Harvard, Cambridge, MA USA

**Keywords:** Vaccines, SARS-CoV-2

## Abstract

The COVID-19 pandemic marks the third coronavirus pandemic this century (SARS-CoV-1, MERS, SARS-CoV-2), emphasizing the need to identify and evaluate conserved immunogens for a pan-sarbecovirus vaccine. Here we investigate the potential utility of a T-cell vaccine strategy targeting conserved regions of the sarbecovirus proteome. We identified the most conserved regions of the sarbecovirus proteome as portions of the RNA-dependent RNA polymerase (RdRp) and Helicase proteins, both of which are part of the coronavirus replication transcription complex (RTC). Fitness constraints suggest that as SARS-CoV-2 continues to evolve these regions may better preserve cross-reactive potential of T-cell responses than Spike, Nucleocapsid, or Membrane proteins. We sought to determine if vaccine-elicited T-cell responses to the highly conserved regions of the RTC would reduce viral loads following challenge with SARS-CoV-2 in mice using a rhesus adenovirus serotype 52 (RhAd52) vector. The RhAd52.CoV.Consv vaccine generated robust cellular immunity in mice and led to significant reductions in viral loads in the nasal turbinates following challenge with a mouse-adapted SARS-CoV-2. These data suggest the potential utility of T-cell targeting of conserved regions for a pan-sarbecovirus vaccine.

## Introduction

In December 2019, SARS-CoV-2 caused an outbreak of severe pneumonia in Wuhan, China marking the third coronavirus epidemic this century^1,2^. Throughout the current pandemic multiple SARS-CoV-2 variants have emerged^3^. The continued development of new variants as well as the recurrent emergence of pathogenic coronaviruses highlights the need for more broadly applicable vaccines^4,5^.

The goal of our study was to focus on the most highly conserved region of the sarbecovirus proteome with the intention of generating broadly protective T-cell responses. Should an outbreak with a novel sarbecovirus occur, such a vaccine could prove useful either as a primary vaccine or as a supplement to suboptimal protection provided by current SARS-CoV-2 vaccines. The vaccine immunogen in the present study is a 1094 amino acid long stretch of ORF1ab comprising the last part of nsp12 (RdRp) and the first part of nsp13 (Helicase), spanning the most conserved contiguous section of the replication transcription complex (RTC)^6–8^ of the sarbecovirus proteome. We term this region CoV.Consv (coronavirus conserved).

Recent data have identified the RTC as a potential broadly protective coronavirus immunogen. Seronegative healthcare workers (SN-HCW) (determined by anti-spike-1 and anti-nucleoprotein neutralization assays) were found to have memory T cells targeting a wider variety of SARS-CoV-2 viral proteins than a pre-pandemic cohort, with the highest frequency of T cells targeting the RTC. Among the SN-HCWs, those with the highest abundance of RTC specific T cells also had an increase in IFI27, a marker used to detect early SARS-CoV-2 infection^9^, but no seroconversion was observed suggesting subclinical SARS-CoV-2 infection^8^. A different study identified that SARS-CoV-2 infected patients with mild symptoms had a higher abundance of CD8^+^ T cells specific for peptides from sequences sharing high similarity with other human CoVs, including two highly conserved epitopes within nsp12^10^. These studies provided further rationale to assess the protective efficacy of the CoV.Consv immunogen.

To test the immunogenicity and protective efficacy of this immunogen, the Wuhan SARS-CoV-2 variant of the CoV.Consv immunogen was expressed by a rhesus adenovirus serotype 52 vector (RhAd52) with the goal of inducing cellular immunity^11,12^. This vaccine was found to induce antigen specific CD8^+^ T-cell responses in BALB/c mice. After challenge with a mouse-adapted SARS-CoV-2^13,14^, RhAd52.CoV.Consv led to significantly reduced viral loads in the upper respiratory tract and a trend towards reduced viral loads in the lower respiratory tract.

## Results

### Development of RhAd52.CoV.Consv vaccine

Our first objective was to identify the most highly conserved contiguous region of the sarbecovirus proteome. We reasoned that highly conserved regions would be evolutionarily constrained, and thus would also be conserved within SARS-CoV-2 as it continues to evolve during the pandemic, while also eliciting responses that would likely cross-react with other sarbecoviruses. To define the most promising conserved regions, we first codon-aligned sequences spanning all reading frames in the full-length proteome from 82 different sarbecoviruses.

**Fig. 1:**
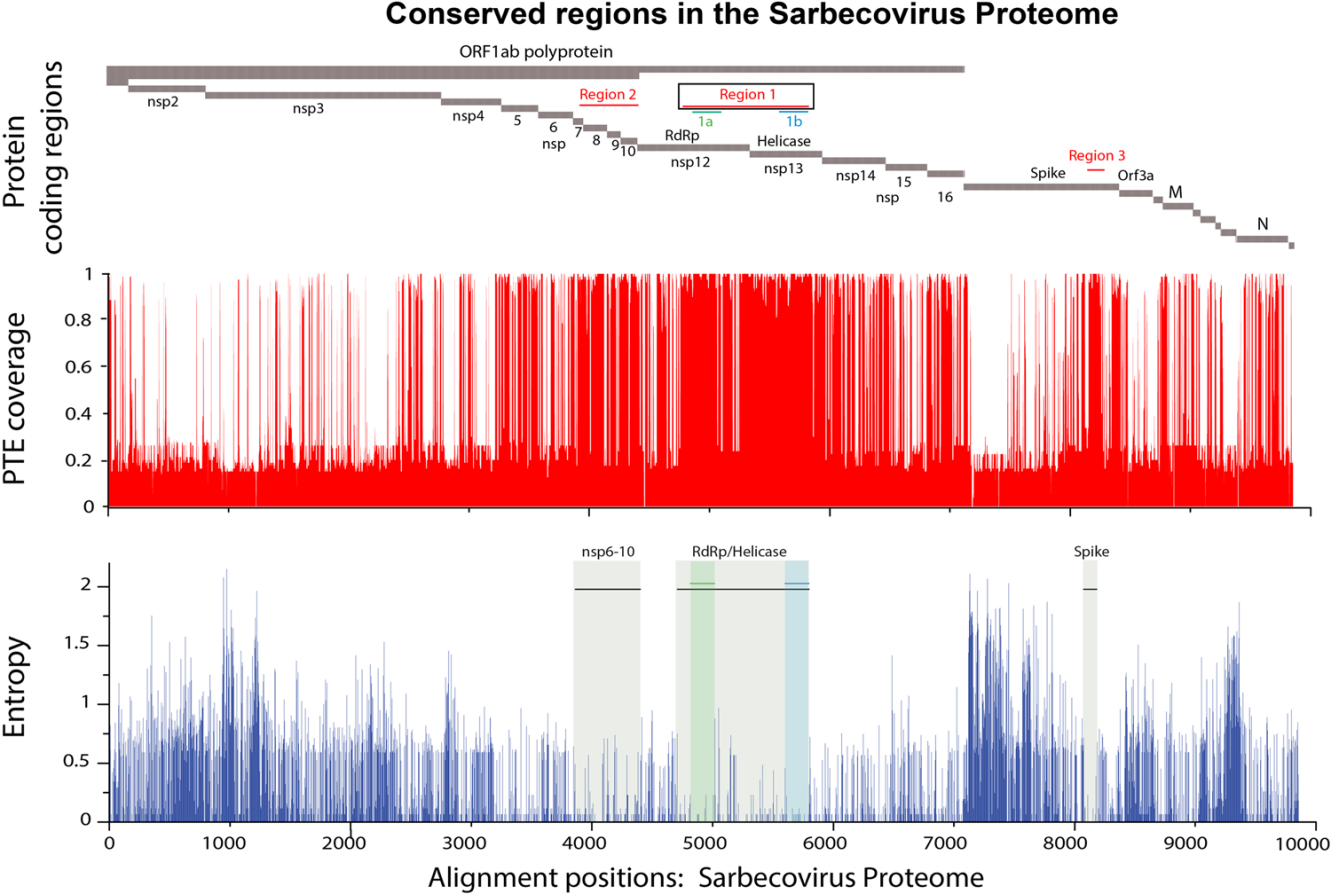
Highly conserved regions identified between multiple sarbecoviruses from bats, pangolin, and human SARS-CoV and SARS-CoV-2. A multiple alignment of 82 sarbecoviruses were selected to capture diversity across the sarbecovirus proteome. The top part of the graph shows where the ORFs and protein coding regions are located within the proteome, the highlighted most conserved regions are labeled Region 1, 2 and 3; Regions 1a and 1b were exceptionally conserved within region 1. The Membrane (M) and Nucleocapsid (N) proteins are also labeled. PTE coverage relative of the aligned proteins by the SARS-CoV-2 Wuhan reference strain (Accession _NC_045512) is indicated in red; a value near 1 means that nearly all of the proteins in the alignment have a perfect match to the 9 amino acid long peptide (PTE) starting in a given position. 17% of the viruses were SARS-CoV-2, so a score of <0.2 indicates that the PTE in the reference strain is commonly matched only among SARS-CoV-2 viruses. Entropy scores for each position in the full proteome alignment are shown in blue in the bottom graph; in this case, lower scores indicate greater conservation, and Regions 1, 2 and 3, are indicated by the beige boxes in this plot, with the most highly conserved stretches highlighted in green and blue.

We identified the regions in the proteome that were most conserved across the viruses in this alignment using two strategies, the entropy at each position in the alignment, and the coverage of Potential T-cell Epitopes (PTEs)^15,16^ in the sarbecovirus alignment by the SARS-CoV-2 pandemic Wuhan reference strain (accession number NC_045512) (Fig. 1). PTEs are defined as all possible linear peptides of 9 amino acids in length in the alignment. As expected, both strategies identified the same three conserved regions (Fig. 1). These included a short region in Spike, 111 amino acids long, S 944–1054 (region 3); a modestly conserved section of the ORF1ab polyprotein beginning in nsp6 and ending in nsp10, 555 amino acids long, ORF1ab 3848–4392 (region 2); and the most conserved and longest stretch, a region spanning part of the RdRp protein (nsp12) and part of the Helicase (nsp13), 1094 amino acids in length, ORF1ab 4692–5785 (region 1). We chose the most conserved region of 1094 amino acids for use in this study (region 1, CoV.Consv), which spanned the region of ORF1ab covering parts of nsp12 and nsp13 and located within the RTC (Fig. 1). Two amino acid mutations, D760A and D761A, were introduced into the RdRp protein for safety to abolish polymerase function^17^.

We hypothesized that this highly conserved region would not be as susceptible as other regions to acquisition of non-synonymous changes within SARS-CoV-2 over time, and this has been observed as variants have emerged^18,19^. For example, the Omicron BA.1 baseline sequence differs in only 1 of 1094 amino acids (0.09%) (nsp12 P323L) from the Wuhan reference sequence in this conserved region, and so 99.1% of the PTEs are perfectly matched between the Omicron BA.1 and the Wuhan reference strain^18^. In contrast, the Omicron BA.1 Spike protein differs from the reference Spike in 39 of 1273 amino acids (3.0%), resulting in only 82.8% perfect PTE matches between the two Spike proteins. This reduction was consistent with the observed reduction in T-cell responses to Omicron BA.1 Spike in vaccinated or convalescent sera^20,21^.

**Fig. 2:**
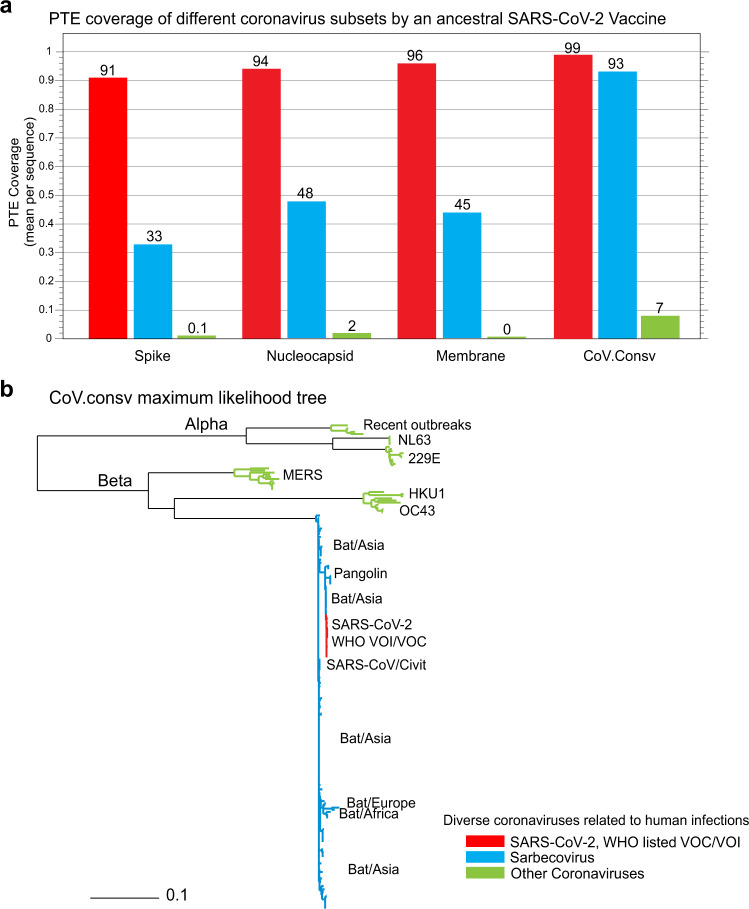
The potential for cross-reactivity between T-cell responses to vaccines based on the S, N, M or the CoV.Consv proteins from a single SARS-CoV-2 Wuhan reference strain vaccine antigen. **a** The average fraction of PTEs (9-mers) shared exactly (PTE coverage) between the putative vaccine antigen and: (i) SARS-CoV-2 pandemic variants (red), (ii) diverse Sarbecoviruses (blue), and (iii) alpha- and betacoronavirses (green) related to those coronaviruses known to infect people, for the S, M, N and the CoV.Consv region. **b** A midpoint rooted phylogenetic tree based on an alignment of the CoV.Consv region using the sequences included in the three different groups used to explore PTE coverage, illustrating the phylogenetic basis of the far greater diversity challenge for vaccines posed by alpha and beta coronaviruses than for sarbecoviruses and SARS-CoV-2.

Alternative T-cell vaccine targets are Membrane (M) and Nucleocapsid (N) as both are highly targeted by T cells^22,23^, making them interesting targets for future vaccine design. Unfortunately, both of these proteins are highly variable among sarbecoviruses (Fig. 1). To assess the relative cross-reactive potential of vaccines targeting proteins of interest, we compared the PTE coverage by S, N, M, and CoV.Consv proteins to three different level of coronavirus diversity. First, we tested the immunogens’ capacity to provide theoretical coverage of SARS-CoV-2 pandemic diversity among SARS-CoV-2 variants of interest and variants of concern (Fig. 2). Second, Sarbecovirus diversity was assessed using an alignment of 162 sequences including 7 human SARS-CoV-1 related sequences isolated from human and civet as well as 155 additional diverse Asian, African, and European sequences sampled from bats and pangolins. A Wuhan reference-based CoV.Consv region antigen provided 93% PTE coverage in the CoV.Consv region, so T-cell responses generated to this region would be predicted to have high cross-reactive potential across sarbecoviruses. In contrast, only 33%, 48%, and 45% of the PTEs were matched for S, N, and M proteins respectively. Finally, other coronavirus data sets were assembled including 42 other coronaviruses known to infect people, and viruses related to these but sampled in non-human hosts (the beta coronaviruses, MERS, OC43, and HKU1, alphacoronaviruses, NL63, and 229E^24^, plus additional coronaviruses that have recently been associated with human infections^25,26^. These viruses are so distant from sarbecoviruses that reliable alignments for phylogenetic reconstructions are not feasible in the M, S, and N proteins, however the CoV.Consv region can be readily aligned and a phylogenetic tree uniting all three data sets, the SARS-CoV-2, sarbecovirus and these other coronaviruses is shown in Fig. 2. We found that almost no PTEs were shared between SARS-CoV-2 and these divergent coronaviruses except for a small number in the CoV.Consv region.

### RhAd52.S.PP suboptimal dose identification

To evaluate the protective efficacy of CoV.Consv, we generated a rhesus adenovirus serotype 52 vector expressing this immunogen (RhAd52.CoV.Consv). Our goal was to identify a suboptimal dose of RhAd52.S.PP to use in combination with the CoV.Consv vaccine in order to model, via a heterologous boost strategy, prior exposure to a spike targeted SARS-CoV-2 vaccine with suboptimal protection. We first performed a dose titration study with adenoviral vectors expressing SARS-CoV-2 Wuhan spike antigens containing a modified furin cleavage site and diproline substitution to stabilize the Spike antigen in a prefusion confirmation (RhAd52.S.PP).

**Fig. 3:**
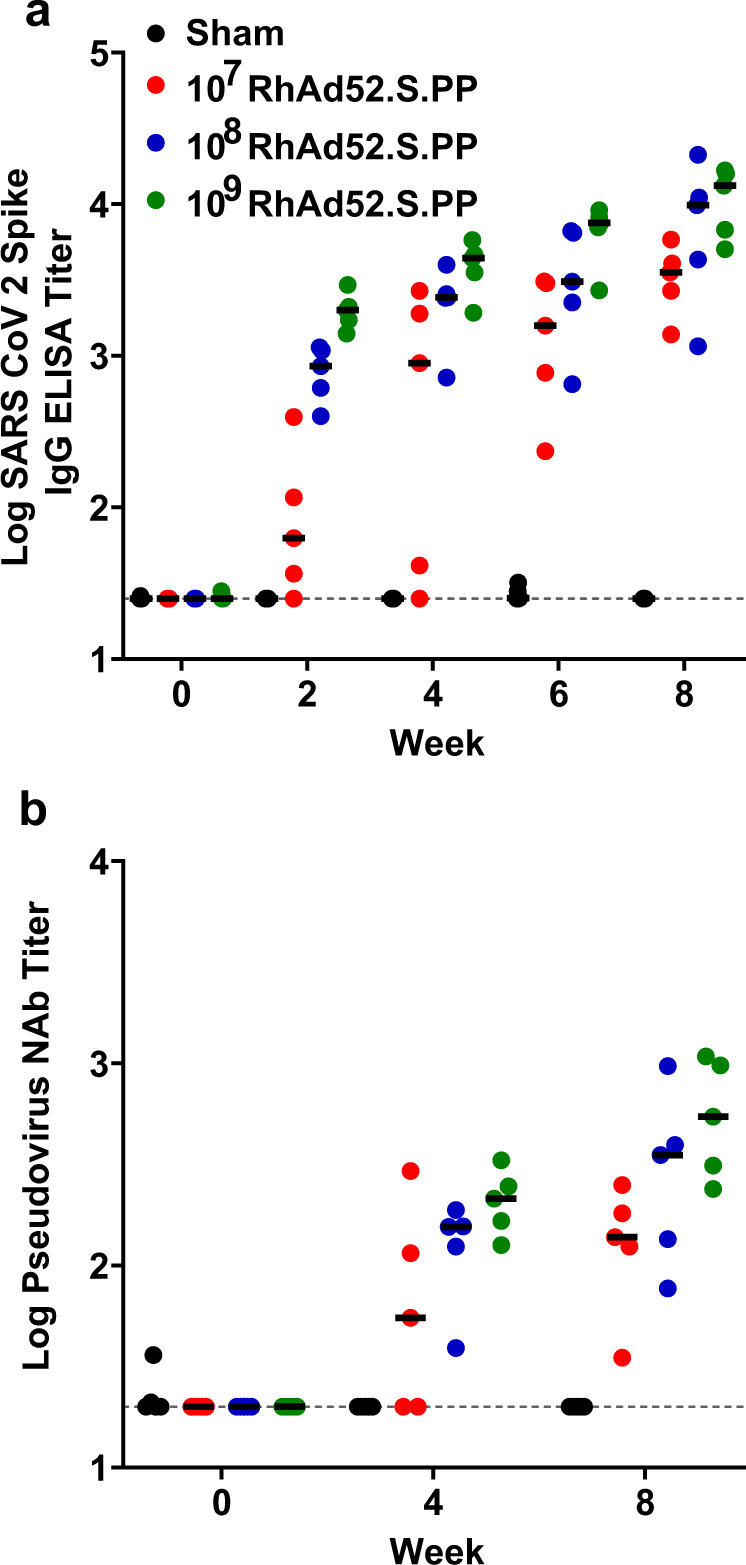
RhAd52.S.PP vaccine titration to generate suboptimal humoral responses. **a**, **b** IgG binding titers against full-length SARS-CoV-2 WH Spike protein (**a**) and neutralizing Ab titers against SARS-CoV-2 WH pseudovirus (**b**) measured over the course of 8 weeks in BALB/c mice vaccinated intramuscularly with RhAd52.S.PP at 10^7^ (red dots), 10^8^ (blue dots), and 10^9^ (green dots) particles per mouse. Each dot represents an individual animal. Median values denoted as black lines.

To identify an appropriate suboptimal dose of RhAd52.S.PP, we vaccinated mice with a single shot of RhAd52.S.PP at 10^7^, 10^8^, and 10^9^ viral particles (vp) per dose. We measured Spike-specific binding antibody titers biweekly for 8 weeks post injection. Interestingly, we found that Spike-specific binding antibodies continued to increase after week 4 with the highest antibody titers at week 8 (Fig. 3a), consistent with recently published work using Ad26^27^. We further investigated this phenomenon by measuring SARS-CoV-2 neutralizing antibodies against the original Wuhan strain. We observed a clear increase in neutralizing antibody titers from week 4 to week 8 for the Wuhan strain (Fig. 3b). This trend was observed for all vaccine doses. We identified 10^7^ vp RhAd52.S.PP as a suboptimal vaccine dose for future experiments^28^.

### Cellular immunity

**Fig. 4:**
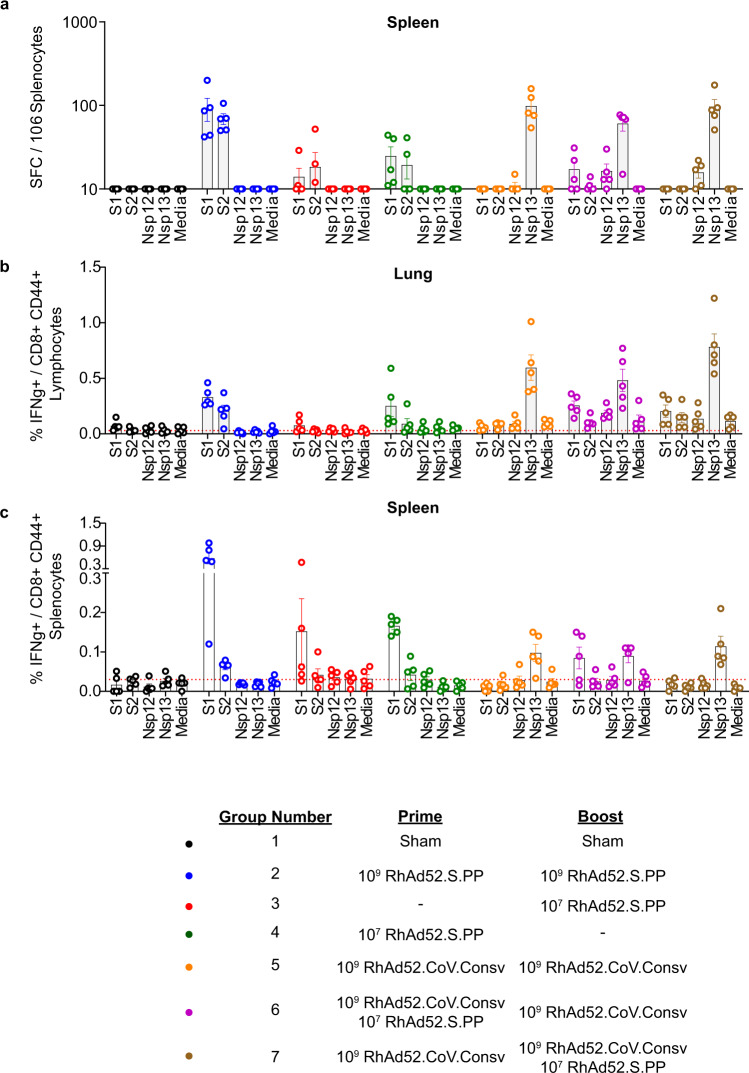
Antiviral cellular immunity elicited by multiple RhAd52.CoV.Consv prime / boost regimens in BALB/c mice. **a**–**c** Antiviral cellular immunity as measured by IFNγ ELISpot (A) and IFNγ-secreting CD8^+^ CD44^+^ T cells in isolated lung (**b**) and spleen (**c**) tissue from BALB/c mice immunized with the designated prime RhAd52 vaccine, boosted with the corresponding RhAd52 vaccine 4 weeks later, and sampled 4 weeks following the boost (8 weeks following the prime immunization). Individual mice are denoted by single dots with bars displaying the mean ± the SEM. Gating strategy and representative flow plots are found in Supplementary Figs. 1–4.

To assess cellular immune responses, BALB/c mice were immunized at week 0 and week 4 with RhAd52 vaccines, and spleen and lungs were collected to evaluate Spike and CoV.Consv specific cellular immunity at week 8. Cellular immunity was evaluated using IFN-γ enzyme-linked immunospot (ELISPOT) assays and intracellular cytokine staining (ICS) assays. Spike-specific cellular immune responses were evaluated by stimulation with S1 and S2 peptide subpools. CoV.Consv responses were studied by stimulation with nsp12 and nsp13 peptide subpools. Using ELISPOT assays, we identified that RhAd52.CoV.Consv vaccination induced cellular responses favoring nsp13 over nsp12 in BALB/c mice (Fig. 4a). ICS assays in the lung showed higher IFN-γ positive CD8^+^ T cells than in the spleen for RhAd52.CoV.Consv vaccination but not for RhAd52.S.PP (Fig. 4b, c, Supplementary Figs. 1–4). This increase in IFN-γ positive CD8^+^ T cells in the lungs compared to spleen was also observed after combination CoV.Consv/S.PP vaccination (Group 6 and 7) in response to both CoV.Consv peptides and spike peptides (Fig. 4b, c, Supplementary Figs. 1–4).

### Protective efficacy

**Fig. 5:**
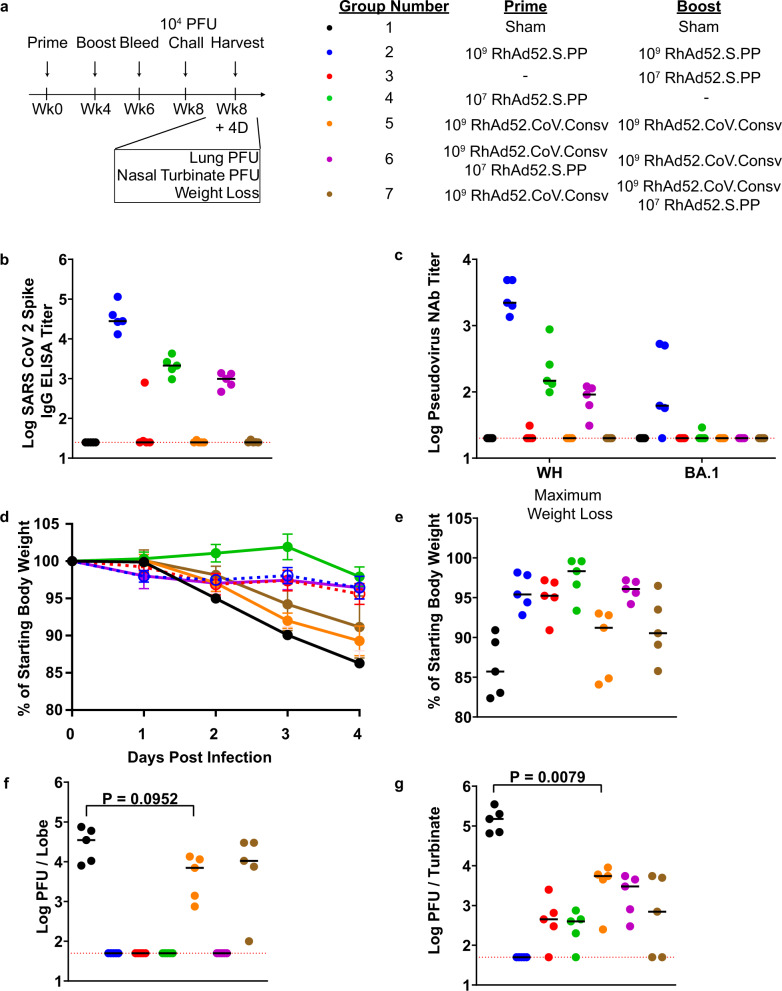
MA10 SARS-CoV-2 challenge of BALB/c mice immunized with RhAd52.CoV.Consv vaccine. **a** Challenge study plan. Groups of BALB/c mice were immunized with different prime/boost regimens of RhAd52.S.PP and CoV.Consv vaccines at week 0 (prime) and week 4 (boost), bled at week 6, and challenged with 10^4^ PFU of mouse-adapted MA10 SARS-CoV-2 at week 8 post-prime. Mouse serum was analyzed for spike-specific binding antibody titers (**b**) and neutralizing antibody titers (**c**) using an ELISA or a pseudovirus neutralization assay, respectively. **d**, **e** Longitudinal weight loss (**d**) and maximum weight loss (**e**) were observed over 4 days post-challenge (Groups 2 and 3 were made dashed to improve viewer clarity). **f**, **g** Lung PFU titers (**f**), and Nasal Turbinate PFU titers (**g**) were measured at day 4 post-challenge for each challenge group. Longitudinal weight loss shown as mean ± the SEM for each prime / boost vaccine group. Individual mice in (**b**, **c**, **e**–**g**) denoted as dots with group median values depicted as a black line. Red dotted lines depict assay limit of detection (**b**, **c**, **f**, **g**). *P* values represent two sided Mann–Whitney tests (**f**, **g**).

In a parallel experiment, we vaccinated mice (Fig. 5) in the same groups as the immunogenicity experiment (Fig. 4). Two weeks post boost we bled the mice to investigate serum antibody levels pre-challenge. Pre-challenge serum was analyzed for Spike-specific antibody titers by ELISA. Antibody responses were detected in the 10^9^ RhAd52.S.PP prime boost group (Group 2) (median 2.8E4 IgG titer), the 10^7^ RhAd52.S.PP 8 week prime (Group 4) (median 2.1E3 IgG titer), and the combination vaccination where 10^7^ RhAd52.S.PP was added at prime (Group 6) (median 9.9E2 IgG titer) (Fig. 5b). Serum was also analyzed using a pseudovirus neutralization assay for the Wuhan and the BA.1 strain of SARS-CoV-2 and confirmed the binding antibody data (Fig. 5c).

At 4 weeks post boost, we challenged vaccinated BALB/c mice with 10^4^ plaque-forming units (PFU) mouse-adapted SARS-CoV-2 Wuhan strain (Fig. 5a). After challenge we tracked weight for 4 days (Fig. 5d) and recorded maximum weight loss per mouse (Fig. 5e). Mice were then sacrificed, and PFU assays were used to quantify the amount of infectious virus in the lungs (Fig. 5f) and nasal turbinate (Fig. 5g). Sham vaccinated mice (Group 1) exhibited severe weight loss with peak mean weight loss of 13.72% (median maximum weight loss 14.29%). Mice vaccinated with 10^9^ RhAd52.S.PP (Group 2) showed a marked reduction in mean weight loss (3.55%) (median maximum weight loss 4.59%) compared to sham vaccinated. RhAd52.CoV.Consv alone (Group 5) achieved only a minimal difference (4.76%) in weight loss compared to the sham group (10.72%). Interestingly, addition of RhAd52.S.PP at week 0 (Group 6) but not week 4 (Group 7) to the two dose RhAd52.CoV.Consv vaccine regiment led to a reduction in weight loss. This observation can likely be attributed to increased spike antibodies over time (Figs. 3, 5b, c).

PFU assays were performed on lungs (Fig. 5f) and nasal turbinates (Fig. 5g) from infected mice at 4 days post infection. Sham vaccinated mice had a median PFU of 3.5E4 per lobe and 1.5E5 in the nasal turbinates. Vaccination with 10^9^ RhAd52.S.PP (Group 2) reduced PFU to undetectable (Limit of Detection: 100 PFU/lobe or nasal turbinate) in the lungs and nasal turbinates. A suboptimal dose of RhAd52.S.PP at either 8 (Group 4) or 4 (Group 3) weeks pre-challenge led to undetectable lung PFU and reduced nasal turbinate PFU. RhAd52.CoV.Consv (Group 5) led to a nonsignificant trend of a 0.5 log reduction of virus in the lungs (median: 7E3 PFU/lobe) and a significant 1.5 log reduction of virus in nasal turbinates (median: 5500 PFU/nasal turbinate) compared to sham controls (*P* = 0.0079). The addition at prime (Group 6) or at boost (Group 7), of a suboptimal RhAd52.S.PP vaccination to the two dose RhAd52.CoV.Consv regimen had a minimal effect.

## Discussion

The three coronavirus epidemics over the past 20 years highlight the need for pan-sarbecovirus vaccines. In this study, we identified and evaluated the most highly conserved region in the sarbecovirus proteome as a candidate vaccine immunogen. We identified a region termed CoV.Consv, which spanned the junction between the SARS-CoV-2 nsp12 and nsp13 proteins as highly conserved among human sarbecoviruses, and more conserved than Membrane, Nucleocapsid, and Spike. RhAd52 vectors expressing CoV.Consv elicited cellular immune responses and led to reduced virus in the upper respiratory tract following mouse-adapted SARS-CoV-2 challenge of BALB/c mice. These data are consistent with prior studies from our laboratory that showed that depletion of CD8^+^ T cells from convalescent rhesus macaques prior to SARS-CoV-2 rechallenge led to increased viral replication in the upper respiratory tract^29^. Future studies could include T-cell depletion experiments following RhAd52.CoV.Consv vaccination.

Previous publications identified the RTC as a highly conserved coronavirus region^8,10^. In one study of seronegative healthcare workers, those with detectable RTC specific T-cell responses were more likely to demonstrate features of abortive SARS-CoV-2 infection^8^. In a separate report, CD8^+^ T cells targeting the RTC were associated with milder COVID-19 disease^10^. Both reports emphasized the need to investigate the RTC as a potential pan-coronavirus immunogen. Our results suggest that these regions have potential as vaccine immunogens for SARS-CoV-2 as well as other sarbecoviruses.

To further investigate T-cell based coronavirus vaccines, use of an animal model with longer disease duration would be desirable. In the mouse-adapted SARS-CoV-2 challenge model, disease naturally begins to resolve after day 4 with virus clearance by day 6 or 7, while human disease extends to about 2 weeks^13,14^. The short timescale of the mouse model is sufficient for observing sterilizing immunity, but reduction in disease severity may be more difficult to observe. Thus, apparent benefits conferred by T-cell responses to the highly conserved regions within the RTC in humans may not be apparent using this animal model system. In the future, alternative models could be explored such as hamsters where disease typically peaks on the order of 7 days post infection^30^. In addition, it may be useful to combine this T-cell focused vaccine with an antibody-based B-cell vaccine.

In conclusion, this CoV.Consv vaccine-elicited T-cell responses in mice and led to a modest but significant reduction of virus in the upper respiratory tract in a mouse-adapted acute model of SARS-CoV-2 infection. A single CoV.Consv immunogen would likely suffice for broad pan-sarbecovirus coverage of this region, but for broader alpha and betacoronavirus coverage, polyvalent designs would likely be essential even for the CoV.Consv region. Combining multivalent Spike immunogens with CoV.Consv may prove useful for candidate pan-sarbecovirus vaccines.

## Methods

### Defining regions of conservation in the sarbecovirus proteome

Our original alignment of sarbecoviruses included 14 SARS-CoV-2 variants, 14 SARS-CoV variants, 48 diverse sarbecovirus isolates from bats, and 6 pangolin sequences, a data set assembled in 2020, that started from the sarbecovirus reference alignment used in Li et al.^31^. We identified the regions of the proteome that were most conserved across the viruses in this alignment using two strategies (Fig. 1). First, we used Shannon entropy as a measure of relative diversity in each position in the full proteome alignment, using the *Entropy* tool at the Los Alamos HIV database (www.hiv.lanl.gov)^32,33^. Next, we used the *Positional Epitope Coverage Assessment Tool* to identify the regions of the proteome that enabled the best coverage of Potential T-cell Epitopes PTEs^15^ in the sarbecovirus alignment by the Wuhan reference variant of the SARS-CoV-2 pandemic (GenBank the reference strain NC_045512, or GISAID variant WIV04, EPI_ISL_402124, which are equivalent). PTEs are defined as all possible linear peptides of 9 amino acids in length in the alignment. Nine amino acids was selected as the peptide length for PTE coverage assessment as it is the most common length of CD8+ T-cell epitopes, and is also the typical length of the core region of CD4+ T-cell epitopes. As expected, both strategies identified precisely the same three highly conserved regions within the alignment, as shown in Fig. 1. The alignment set included some subsets of highly related viruses, like the human SARS-CoV-2 subset, and pangolin virus subset; the proportion of these groups will of course impact the values of the entropy and PTE coverage scores. However, as a codon-aligned full-length genome alignment was used, the composition of variant strains was identical across all protein coding regions, thus the relative scores in the different protein regions enables a meaningful comparison, and it was these relative values across the proteome that were used for defining conserved regions. PTE scores approaching 1 dominate in the conserved regions we defined, so these regions were extremely highly conserved, including among the diverse variants isolated from bats. In contrast, PTE scores of ≤0.17 reflect 9-mers that match the Wuhan reference sequence only among the 14 SARS-CoV-2 viruses included in the alignment.

An extended alignment of the CoV.Consv region generated in 2022 was used to create the tree in Fig. 2, including a representative sequence of each WHO variant of interest or concern (VOI and VOC) and Omicron variant noted to be important for continued monitoring through July 2022 (https://www.who.int/activities/tracking-SARS-CoV-2-variants). The SARS-CoV-2 alignments are available through the Los Alamos National Laboratory download page at GISAID. An additional 162 sarbecovirus sequences were added based on literature^34–40^ and GenBank searches, selected to be representative of diversity, and an additional 44 alpha- and betacoronavirus sequences were added based on BLAST searches of coronavirus from human infections that were down selected to have complete sequences spanning CoV.Consv, S, M and N proteins and were representative of sampled diversity among similar sequences. This alignment is available, and the tree shown in Fig. 2 was generated using IQ-tree 1.6.11^41^ based on the CoV.Consv region using protein sequences, with a best fit model of LG + F + R3 and midpoint rooting, run via the Los Alamos HIV database IQ-tree web interface and visualized using their Rainbow tree tool.

### RhAd52 vectors

RhAd52.CoV.Consv vaccines were generated from region 1 of the SARS-CoV-2 genome (Fig. 1). The sequences were codon optimized and synthesized through Thermo Fisher Scientific Geneart. Replication incompetent, RhAd52 vectors with E1/E3 regions deleted were grown in HEK 293B-55K.TetR cells^42^. The E1 region was replaced by the CoV.Consv immunogen. All vectors were sequenced and tested for infectivity before use.

### Animals and study design

Female BALB/c mice (The Jackson Laboratory) were randomly allocated to groups. Mice received RhAd52 vectors expressing the SARS-CoV-2 prefusion spike protein with diproline mutation, CoV.Consv immunogen, a combination of the two, or sham controls (*N* = 5 per group). Animals received a prime and boost immunization of either 10^7^ or 10^9^ viral particles (VPs) of RhAd52 vectors by the intramuscular route without adjuvant. All Adenoviral vector injections were 10^9^ VPs unless otherwise stated. Peripheral blood was collected via the submandibular route to isolate serum for immunologic assays. Spleen and lungs were collected and processed for cellular assays. For viral challenge, mice were administered 1 × 10^4^ PFU MA10 SARS-CoV-2 in a volume of 50 μL via the intranasal route^13,14^. Following challenge, body weights were assessed daily. Animals were euthanized on day 4 post-challenge for viral outgrowth assays. All animal studies were conducted in compliance with all relevant local, state and federal regulations and were approved by the Beth Israel Deaconess Medical Center and University of North Carolina at Chapel Hill Institutional Animal Care and Use Committees.

### SARS-CoV-2 Spike ELISA

S binding antibodies were assessed as follows: 96-well plates (Thermo Fisher Scientific) were coated with 1 µg/ml of SARS-CoV-2 spike protein (Sino Biological) in 1X DPBS and incubated at 4 °C overnight. After incubation, plates were washed once with wash buffer (0.05% Tween 20 in 1X DPBS) and blocked with 350 µL of Casein per well for 2–3 h at room temperature after which block solution was discarded and plates were blotted dry. Three-fold serial dilutions of mouse serum in casein block were added to wells and plates were incubated for 1 h at room temperature. Plates were washed three times before adding rabbit anti-mouse IgG HRP (Jackson ImmunoResearch) diluted 1:1000 in casein to each well. After 1 h incubation in the dark, plates were washed three times. Next, 100 µL of SeraCare KPL TMB SureBlue Start solution was added to each well. After 3 m 30 s, 100uL of SeraCare KPL TMB Stop solution was added to halt development. Using a Versamax microplate reader, absorbance at 450 nm was recorded. Raw OD values were transferred to Graphpad Prism where a standard curve was interpolated using a sigmoidal four-parameter logistic (4PL) fit. ELISA endpoint titers were quantified using the interpolation function to calculate reciprocal serum dilution at which OD value equal 0.2.

### Pseudovirus neutralization assay

A SARS-CoV-2 pseudovirus expressing a luciferase reporter gene was generated as follows: The packaging construct psPAX2 (AIDS Resource and Reagent Program), luciferase reporter plasmid pLenti-CMV Puro-Luc (Addgene) and S protein expressing pcDNA3.1-SARS-CoV-2 S.dCT were co-transfected into HEK293T cells using lipofectamine 2000 (Thermo Fisher Scientific). Pseudotype virus was purified from supernatants collected 48 h later using a 0.45-µm filter. To determine the neutralization activity of the antisera from vaccinated mice, HEK293T-hACE2 target cells were seeded in 96-well tissue culture plates at a density of 2.0 × 10^4^ cells per well and incubated overnight. Three-fold serial dilutions of heat-inactivated serum were prepared and mixed with 60 µl of pseudovirus. The mixture was incubated at 37 °C for 1 h before adding to HEK293T-hACE2 cells. After 48 h, cells were lysed in Steady-Glo Luciferase (Promega) according to the manufacturer’s instructions. SARS-CoV-2 neutralizing antibody titers were defined as the sample dilution at which a 50% reduction in relative light units was observed relative to the average of the virus control wells.

### PFU assay

Lung viral titers were determined by plaque assay. Briefly, right caudal lung lobes were homogenized in 1 mL PBS using glass beads and serial dilutions of the clarified lung homogenates were added to a monolayer of Vero E6 cells and overlayed with a solution of 0.8% agarose and media. After 3 days, plaques were visualized via staining with Neutral Red dye and counted.

### ELIspot

ELIspot plates were coated with rat anti-mouse IFN-γ monoclonal antibody (BD Biosciences 554410) diluted 1:100 at a final concentration of 500 ng per well overnight at 4 °C. Plates were washed with DPBS containing 0.25% Tween 20, and blocked with R10 media (RPMI with 10% FBS and 1% penicillin-streptomycin) for 1 h at 37 °C. The Spike 1, Spike 2, nsp12, and nsp13 peptide pools contain 15 amino acid peptides overlapping by 11 amino acids that span the protein sequence and reflect the N- and C- terminal halves of the protein, respectively. Spike 1 and 2 as well as nsp12 and nsp13 were prepared at a concentration of 2 µg per well with 500,000 cells per well added. The peptides and cells were incubated for 18–24 h at 37 °C. All steps following this incubation were performed at room temperature. The plates were washed with coulter buffer and incubated for 2 h with Rat anti-mouse IFN-γ Biotin (diluted 1:200) from BD Biosciences (551216) (375 ng/well). Plates were washed again and then incubated with Streptavidin-alkaline phosphatase antibody from Southern Biotechnology (7105–04) (20 µL/well). The third wash was followed by the addition of Nitor-blue Tetrazolium Chloride/5-bromo-4-chloro 3 ‘indolyl phosphate p-toluidine salt (NBT/BCIP chromagen) from Thermo fisher Scientific (34042) for 7 min. Chromagen was discarded and plates were washed with water and dried in a dark place for 24 h. Plates were scanned and counted on a Cellular Technologies Limited Immunospot Analyzer.

### Intracellular cytokine staining assay

Lungs were places into Miltenyi gentleMACS C-Tubes (130-093-237) containing 4 mL R10 and 1 mL 5x digestion buffer (16.5 mL R10, 100 mg Type IV Collagenase). Lungs were processed using the Miltenyi gentleMACS Dissociator with heating (130-093-235) on “m_lung_01_02” program. Lung cells were then passed through a 70 µm filter. Cells were spun down for 5 min at 1400 rpm. Cells were resuspended in 3 mL 1x ACK Lysing Buffer (gibco A10492-01) for 3 min to lyse red blood cells. ACK was neutralized with 20 mL R10. Cells were spun down for 5 min at 1400 rpm. Lung cells were then resuspended in 10 mL R10 and run through 30 µm filter and were then treated as splenocytes for ICS. Splenocytes were resuspended in 100 µL of R10 media. Each sample was incubated with mock (100 µL of R10; background control), Spike 1, Spike 2, nsp12, nsp13 peptide pools (2 µg/mL), or Leukocyte Activation Cocktail with BD GolgiPlug from BD Biosciences (550583) (positive control) and incubated at 37 °C for 1.5 h. After incubation, 0.2 µL of GolgiStop and 0.2 µL of GolgiPlug in 50 µL of R10 was added to each well and incubated at 37 °C for 5 h and then held at 4 °C overnight. The following day, cells were washed twice with MACS Buffer, stained with Aqua live/dead dye and then stained with predetermined titers of mAbs against CD8a (clone 53–6.7; BUV395), CD3 (clone 17A2; BUV737), CD103 (clone 2E7; BV605), CD44 (clone IM7; BV711), CD62L (clone MEL-14; Alexa Fluor 488), CD4 (clone RM4–5; PE/Dazzle 594), and CD69 (clone H1.2F3; PE-Cy7) (all antibodies diluted 1:100) for 1 h. Cells were then washed twice with MACS buffer and incubated with 100 µL of BD Cytofix/CytoPerm Fixation/Permeabilization solution for 15 min. Cells were then washed twice with 1x Perm Wash buffer (BD Perm/WashTM Buffer 10x in CytoFix/CytoPerm Fixation/ Permeabilization kit diluted with MilliQ water and passed through 0.22 µm filter) and stained intracellularly with mAbs against IFN-γ (clone XMG1.2; BV421), IL-2 (clone JES6-5H4; PE), IL-4 (clone 11B11; APC), and TNFα (clone MP6-XT22; Alexa Fluor 700) (all antibodies diluted 1:100) for 1 h. Cells were then washed twice with 1X Perm Wash buffer and washed once with MACS buffer then fixed with 250 µL of freshly prepared 2% formaldehyde. Fixed cells were analyzed by BD LSRII system.

### Reporting summary

Further information on research design is available in the Nature Research Reporting Summary linked to this article.

## Supplementary information

Supplemental Figures

REPORTING SUMMARY

## Data Availability

Authors can confirm that all relevant data are included in the paper.

## References

[1] Zhou P (2020). A pneumonia outbreak associated with a new coronavirus of probable bat origin. Nature.

[2] Li Q (2020). Early transmission dynamics in Wuhan, China, of novel coronavirus—infected pneumonia. N. Engl. J. Med..

[3] Abdool Karim SS, de Oliveira T (2021). New SARS-CoV-2 variants—clinical, public health, and vaccine implications. N. Engl. J. Med..

[4] Ksiazek TG (2003). A novel coronavirus associated with severe acute respiratory syndrome. N. Engl. J. Med..

[5] Alagaili AN (2014). Middle east respiratory syndrome coronavirus infection in dromedary camels in Saudi Arabia. mBio.

[6] Romano M, Ruggiero A, Squeglia F, Maga G, Berisio R (2020). A structural view of SARS-CoV-2 RNA replication machinery: RNA synthesis, proofreading and final capping. Cells.

[7] Chen J (2020). Structural basis for helicase-polymerase coupling in the SARS-CoV-2 replication-transcription complex. Cell.

[8] Swadling, L. et al. Pre-existing polymerase-specific T cells expand in abortive seronegative SARS-CoV-2. *Nature*, 10.1038/s41586-021-04186-8 (2021). 10.1038/s41586-021-04186-8 PMC8732273 34758478

[9] Gupta RK (2021). Blood transcriptional biomarkers of acute viral infection for detection of pre-symptomatic SARS-CoV-2 infection: a nested, case-control diagnostic accuracy study. Lancet Microbe.

[10] Mallajosyula V (2021). CD8+ T cells specific for conserved coronavirus epitopes correlate with milder disease in patients with COVID-19. Sci. Immunol..

[11] Tatsis N, Ertl HCJ (2004). Adenoviruses as vaccine vectors. Mol. Ther..

[12] Barouch DH, Nabel GJ (2005). Adenovirus vector-based vaccines for human immunodeficiency virus type 1. Hum. gene Ther..

[13] Dinnon KH (2020). A mouse-adapted model of SARS-CoV-2 to test COVID-19 countermeasures. Nature.

[14] Leist SR (2020). A mouse-adapted SARS-CoV-2 induces acute lung injury and mortality in standard laboratory mice. Cell.

[15] Fischer W (2007). Polyvalent vaccines for optimal coverage of potential T-cell epitopes in global HIV-1 variants. Nat. Med..

[16] Theiler J (2016). Epigraph: a vaccine design tool applied to an HIV therapeutic vaccine and a Pan-Filovirus vaccine. Sci. Rep..

[17] Gao Y (2020). Structure of the RNA-dependent RNA polymerase from COVID-19 virus. Science.

[18] Wang L, Cheng G (2022). Sequence analysis of the emerging SARS-CoV-2 variant Omicron in South Africa. J. Med. Virol..

[19] Banu TA (2021). Genome sequencing of the SARS-CoV-2 Delta (B.1.617.2) variant of concern detected in Bangladesh. Microbiol. Resour. Announcements.

[20] Liu, J. et al. Vaccines elicit highly conserved cellular immunity to SARS-CoV-2 omicron. *Nature*10.1038/s41586-022-04465-y (2022). 10.1038/s41586-022-04465-y PMC8930761 35102312

[21] Keeton, R. et al. T cell responses to SARS-CoV-2 spike cross-recognize omicron. *Nature*10.1038/s41586-022-04460-3 (2022). 10.1038/s41586-022-04460-3 PMC8930768 35102311

[22] Tarke A (2021). Comprehensive analysis of T cell immunodominance and immunoprevalence of SARS-CoV-2 epitopes in COVID-19 cases. Cell Rep. Med..

[23] Vidal SJ (2021). Correlates of neutralization against SARS-CoV-2 variants of concern by early pandemic sera. J. Virol..

[24] Cui J, Li F, Shi ZL (2019). Origin and evolution of pathogenic coronaviruses. Nat. Rev. Microbiol..

[25] Vlasova AN (2022). Animal alphacoronaviruses found in human patients with acute respiratory illness in different countries. Emerg. Microbes Infect..

[26] King A (2021). Two more coronaviruses may infect people. Science.

[27] Mahrokhian SH (2022). Durability and expansion of neutralizing antibody breadth following Ad26.COV2.S vaccination of mice. npj Vaccines.

[28] Tostanoski LH (2021). Protective efficacy of Rhesus Adenovirus COVID-19 Vaccines against Mouse-Adapted SARS-CoV-2. J. Virol..

[29] McMahan K (2021). Correlates of protection against SARS-CoV-2 in rhesus macaques. Nature.

[30] Tostanoski LH (2020). Ad26 vaccine protects against SARS-CoV-2 severe clinical disease in hamsters. Nat. Med..

[31] Li X (2020). Emergence of SARS-CoV-2 through recombination and strong purifying selection. Sci. Adv..

[32] Reza, F. M. *An introduction to information theory*. 76-124 (Courier Corporation, 1994).

[33] Korber BT (1994). Genetic differences between blood- and brain-derived viral sequences from human immunodeficiency virus type 1-infected patients: evidence of conserved elements in the V3 region of the envelope protein of brain-derived sequences. J. Virol..

[34] Alkhovsky, S. et al. SARS-like Coronaviruses in Horseshoe Bats (Rhinolophus spp.) in Russia, 2020. *Viruses* **14**, 113 (2022). 10.3390/v14010113 PMC8779456 35062318

[35] Delaune D (2021). A novel SARS-CoV-2 related coronavirus in bats from Cambodia. Nat. Commun..

[36] Ge X-Y (2013). Isolation and characterization of a bat SARS-like coronavirus that uses the ACE2 receptor. Nature.

[37] Song H-D (2005). Cross-host evolution of severe acute respiratory syndrome coronavirus in palm civet and human. Proc. Natl Acad. Sci..

[38] Temmam S (2022). Bat coronaviruses related to SARS-CoV-2 and infectious for human cells. Nature.

[39] Wells, H. L. et al. The evolutionary history of ACE2 usage within the coronavirus subgenus Sarbecovirus. *Virus Evol.* **7**, 10.1093/ve/veab007 (2021). 10.1093/ve/veab007 PMC7928622 33754082

[40] Wu, Z. et al. A comprehensive survey of bat sarbecoviruses across China in relation to the origins of SARS-CoV and SARS-CoV-2. *Natl Sci. Rev.*10.1093/nsr/nwac213 (2022). 10.1093/nsr/nwac213 PMC10325003 37425654

[41] Trifinopoulos J, Nguyen L-T, von Haeseler A, Minh BQ (2016). W-IQ-TREE: a fast online phylogenetic tool for maximum likelihood analysis. Nucleic Acids Res..

[42] Abbink P (2007). Comparative seroprevalence and immunogenicity of six rare serotype recombinant adenovirus vaccine vectors from subgroups B and D. J. Virol..

