# Supplementary material for: Immunogenicity and protective efficacy of a rhesus adenoviral vaccine targeting conserved COVID-19 replication transcription complex
Source: NPJ Vaccines. 2022 Oct 27;7:125. doi: 10.1038/s41541-022-00553-2 (PMC9610341; doi:10.1038/s41541-022-00553-2)
Supplement: Supplementary file 1 — Supplemental Figures [file 41541_2022_553_MOESM1_ESM.pdf]

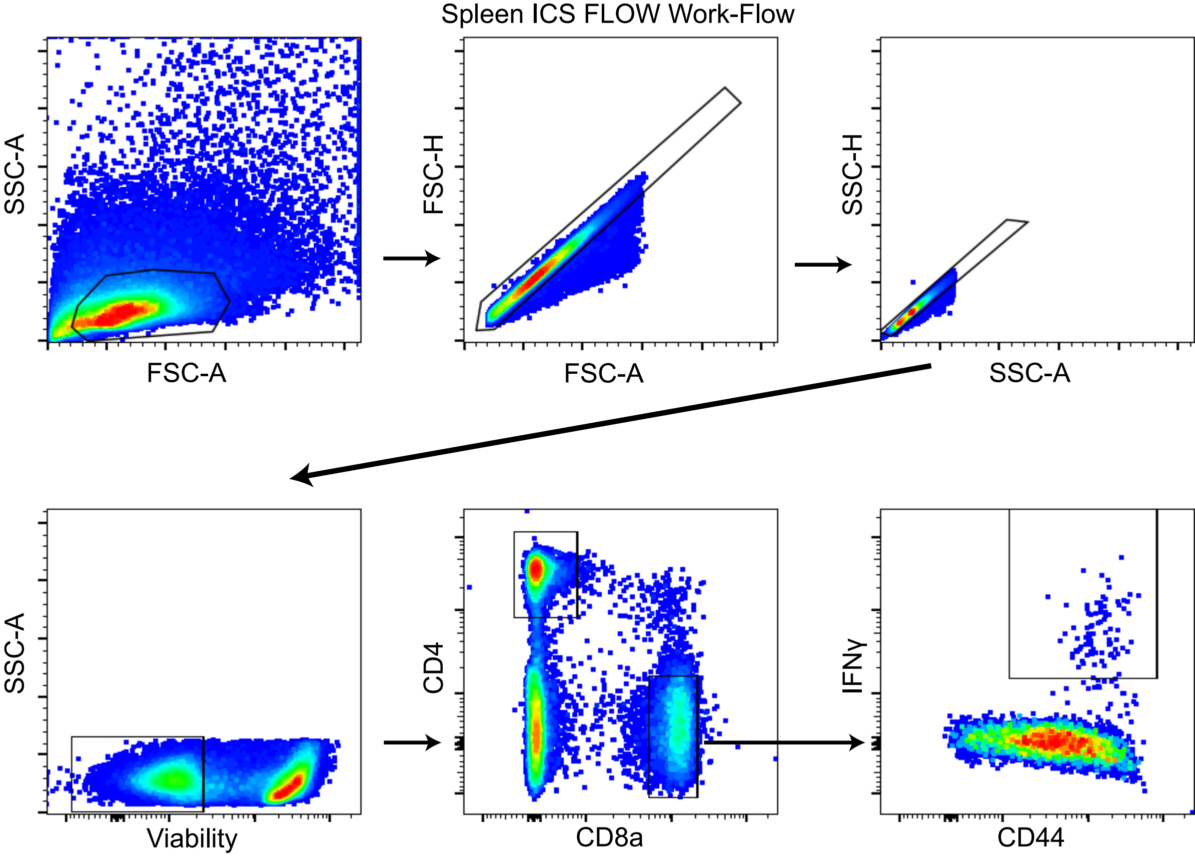

Supplementary Figure 1: Spleen Intracellular Cytokine FLOW Panel Workflow. Gating strategy for identifying IFNγ-secreting CD8+ CD44+ T cells in the spleen. This gating strategy was used to collect data for figure 3c.

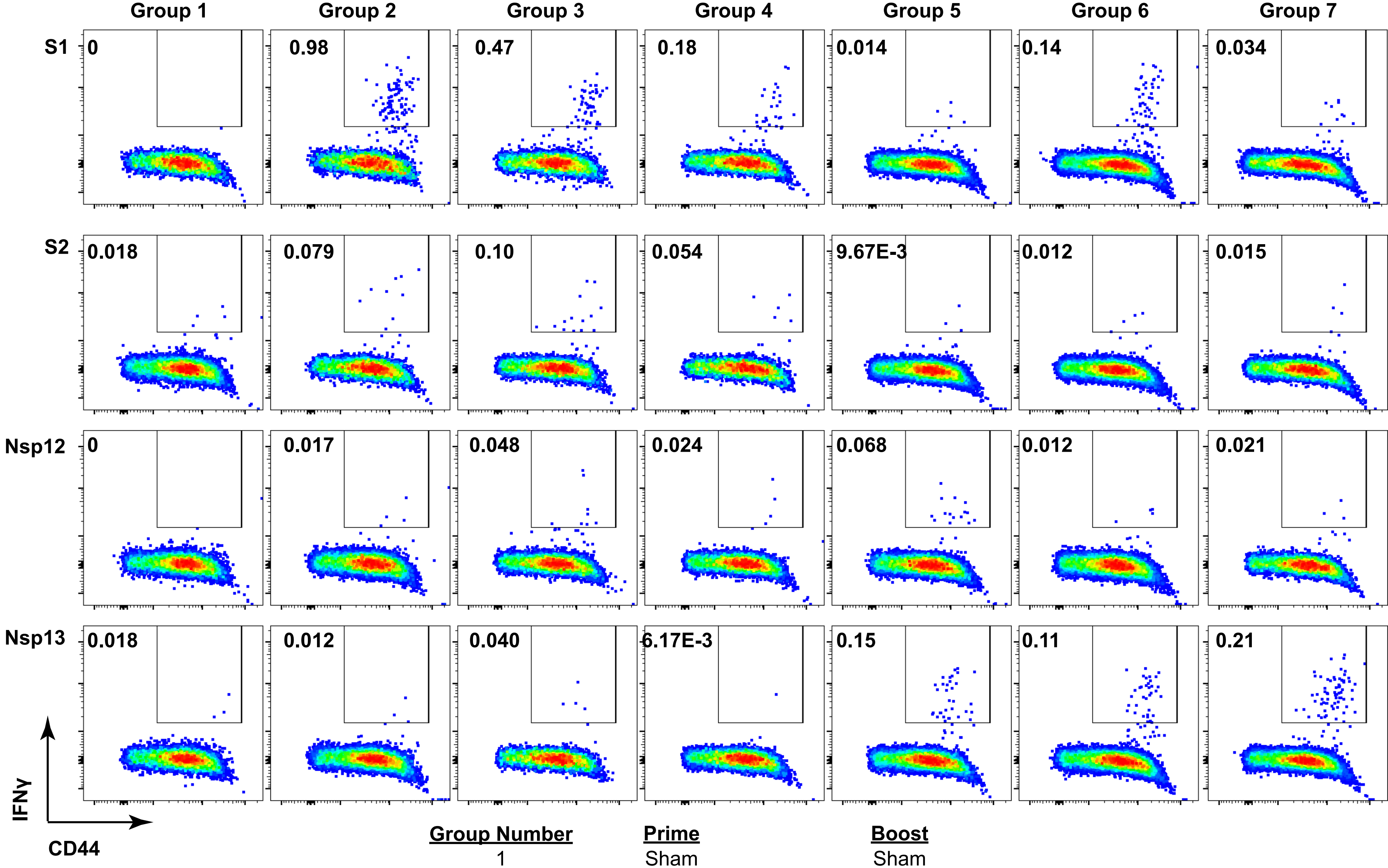

Group Number	Prime	Boost
1	Sham	Sham
2	10^9 RhAd52.S.PP	10^9 RhAd52.S.PP
3	-	10^7 RhAd52.S.PP
4	10^7 RhAd52.S.PP	-
5	10^9 RhAd52.CoV.Consv	10^9 RhAd52.CoV.Consv
6	10^9 RhAd52.CoV.Consv<br>10^7 RhAd52.S.PP	10^9 RhAd52.CoV.Consv
7	10^9 RhAd52.CoV.Consv	10^9 RhAd52.CoV.Consv<br>10^7 RhAd52.S.PP

Supplementary Figure 2: Representative data for spleen intracellular cytokine stain assay. Representative flow plots from each group (x-axis) under each stim condition (y-axis) are shown from figure 3c. The individual plot y-axes shows IFNγ signal and the individual plot x-axes shows CD44 signal. Numbers in each plot represent % events in the shown gate.

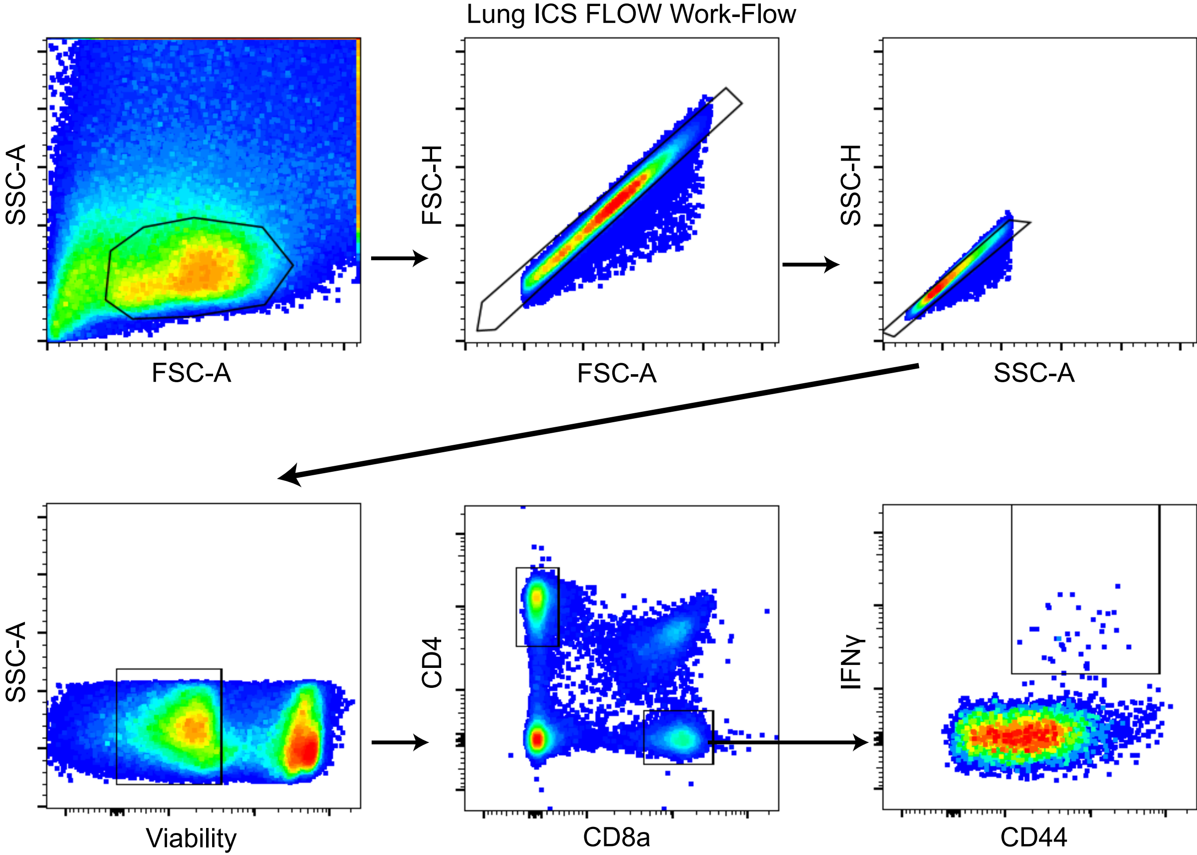

Supplementary Figure 3: Lung Intracellular Cytokine FLOW Panel Workflow.
Gating strategy for identifying IFNγ-secreting CD8+ CD44+ T cells in the lungs. This gating strategy was used to collect data for figure 3b.

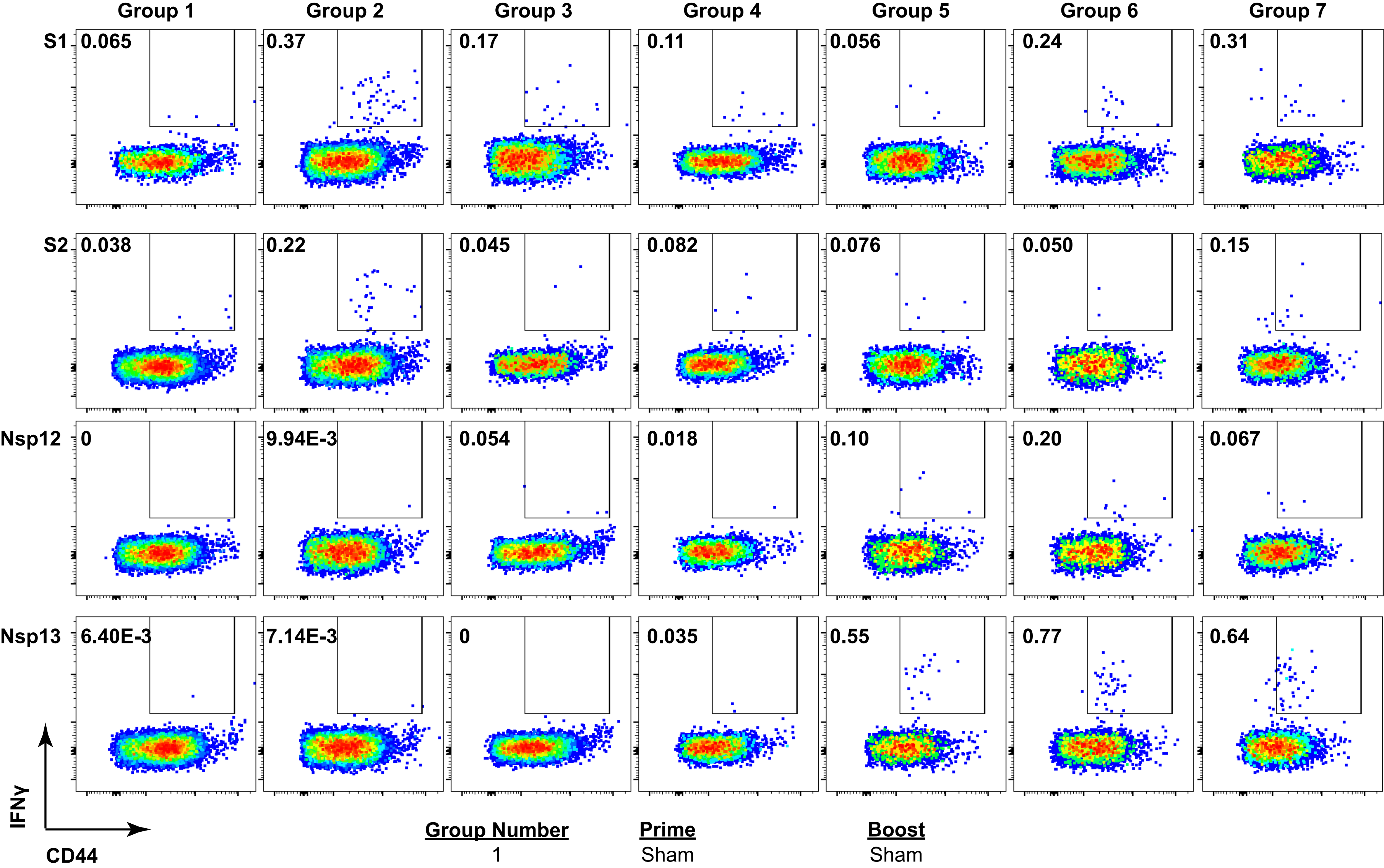

Group Number	Prime	Boost
1	Sham	Sham
2	10^9 RhAd52.S.PP	10^9 RhAd52.S.PP
3	-	10^7 RhAd52.S.PP
4	10^7 RhAd52.S.PP	-
5	10^9 RhAd52.CoV.Consv	10^9 RhAd52.CoV.Consv
6	10^9 RhAd52.CoV.Consv<br>10^7 RhAd52.S.PP	10^9 RhAd52.CoV.Consv
7	10^9 RhAd52.CoV.Consv	10^9 RhAd52.CoV.Consv<br>10^7 RhAd52.S.PP

Supplementary Figure 4: Representative data for lung intracellular cytokine stain assay. Representative flow plots from each group (x-axis) under each stim condition (y-axis) are shown from figure 3b. The individual plot y-axes shows IFNγ signal and the individual plot x-axes shows CD44 signal. Numbers in each plot represent % events in the shown gate.
