# Supplementary material for: Immunogenicity and protective efficacy of a rhesus adenoviral vaccine targeting conserved COVID-19 replication transcription complex
Source: NPJ Vaccines. 2022 Oct 27;7:125. doi: 10.1038/s41541-022-00553-2 (PMC9610341; doi:10.1038/s41541-022-00553-2)
Supplement: Supplementary file 2 — REPORTING SUMMARY [file 41541_2022_553_MOESM2_ESM.pdf]

nature portfolio

Corresponding author(s): Dan H. Barouch

Last updated by author(s): Aug 15, 2022

Reporting Summary

Nature Portfolio wishes to improve the reproducibility of the work that we publish. This form provides structure for consistency and transparency in reporting. For further information on Nature Portfolio policies, see our Editorial Policies and the Editorial Policy Checklist.

Statistics

For all statistical analyses, confirm that the following items are present in the figure legend, table legend, main text, or Methods section.

n/a	Confirmed	
☐	☒	The exact sample size (n) for each experimental group/condition, given as a discrete number and unit of measurement
☐	☒	A statement on whether measurements were taken from distinct samples or whether the same sample was measured repeatedly
☐	☒	The statistical test(s) used AND whether they are one- or two-sided<br>*Only common tests should be described solely by name; describe more complex techniques in the Methods section.*
☒	☐	A description of all covariates tested
☐	☒	A description of any assumptions or corrections, such as tests of normality and adjustment for multiple comparisons
☐	☒	A full description of the statistical parameters including central tendency (e.g. means) or other basic estimates (e.g. regression coefficient) AND variation (e.g. standard deviation) or associated estimates of uncertainty (e.g. confidence intervals)
☐	☒	For null hypothesis testing, the test statistic (e.g. F, t, r) with confidence intervals, effect sizes, degrees of freedom and P value noted<br>*Give P values as exact values whenever suitable.*
☒	☐	For Bayesian analysis, information on the choice of priors and Markov chain Monte Carlo settings
☒	☐	For hierarchical and complex designs, identification of the appropriate level for tests and full reporting of outcomes
☒	☐	Estimates of effect sizes (e.g. Cohen's d, Pearson's r), indicating how they were calculated

Our web collection on statistics for biologists contains articles on many of the points above.

Software and code

Policy information about availability of computer code

Data collection	Open source software used in these studies include: Entropy tool (Los Alamos HIV database (www.hiv.lanl.gov)), Positional Epitope Coverage Assessment Tool, SARS-CoV-2 alignments available through Los Alamos National Laboratory download page at GISAID.
Data analysis	Microsoft office Excel 2013, Graphpad Prism 9.1.2

For manuscripts utilizing custom algorithms or software that are central to the research but not yet described in published literature, software must be made available to editors and reviewers. We strongly encourage code deposition in a community repository (e.g. GitHub). See the Nature Portfolio guidelines for submitting code & software for further information.

Data

Policy information about availability of data

All manuscripts must include a data availability statement. This statement should provide the following information, where applicable:

- Accession codes, unique identifiers, or web links for publicly available datasets
- A description of any restrictions on data availability
- For clinical datasets or third party data, please ensure that the statement adheres to our policy

Data availability statement included. All data are available in the manuscript. Correspondence and requests for materials should be addressed to D.H.B. (dbarouch@bidmc.harvard.edu)

Human research participants

Policy information about studies involving human research participants and Sex and Gender in Research.

Reporting on sex and gender	No studies involving human participants were used.
Population characteristics	*Describe the covariate-relevant population characteristics of the human research participants (e.g. age, genotypic information, past and current diagnosis and treatment categories). If you filled out the behavioural & social sciences study design questions and have nothing to add here, write "See above."*
Recruitment	*Describe how participants were recruited. Outline any potential self-selection bias or other biases that may be present and how these are likely to impact results.*
Ethics oversight	*Identify the organization(s) that approved the study protocol.*

Note that full information on the approval of the study protocol must also be provided in the manuscript.

Field-specific reporting

Please select the one below that is the best fit for your research. If you are not sure, read the appropriate sections before making your selection.

☒ Life sciences ☐ Behavioural & social sciences ☐ Ecological, evolutionary & environmental sciences

For a reference copy of the document with all sections, see nature.com/documents/nr-reporting-summary-flat.pdf

Life sciences study design

All studies must disclose on these points even when the disclosure is negative.

Sample size	We have found that groups of 5 animals are suitable to reach statistical significance.
Data exclusions	No data was excluded or censored from the study.
Replication	ELISAs and ELIspots were conducted in duplicate. Technical replicates were similar.
Randomization	Female BALB/c mice were randomly allocated to groups.
Blinding	N/A

Reporting for specific materials, systems and methods

We require information from authors about some types of materials, experimental systems and methods used in many studies. Here, indicate whether each material, system or method listed is relevant to your study. If you are not sure if a list item applies to your research, read the appropriate section before selecting a response.

Materials & experimental systems

n/a	Involved in the study	
☐	☒	Antibodies
☐	☒	Eukaryotic cell lines
☒	☐	Palaeontology and archaeology
☐	☒	Animals and other organisms
☒	☐	Clinical data
☒	☐	Dual use research of concern

Methods

n/a	Involved in the study	
☒	☐	ChIP-seq
☐	☒	Flow cytometry
☒	☐	MRI-based neuroimaging

Antibodies

Antibodies used	For ELISA assays: rabbit anti-mouse IgG HRP (Jackson ImmunoResearch). For ELIspot assays: rat anti-mouse IFN-γ monoclonal antibody (BD Biosciences 554410), Rat anti-mouse IFN-γ Biotin from BD Biosciences (551216), Streptavidin-alkaline phosphatase antibody from Southern Biotechnology (7105-04). For Intracellular Cytokine assays: monoclonal antibodies against CD8a (clone 53-6.7; BUV395), CD3 (clone 17A2; BUV737), CD103 (clone 2E7; BV605), CD44 (clone IM7; BV711), CD62L (clone MEL-14; Alexa Fluor

	488), CD4 (clone RM4-5; PE/Dazzle 594), CD69 (clone H1.2F3; PE-Cy7), IFN-γ (clone XMG1.2; BV421), IL-2 (clone JES6-5H4; PE), IL-4 (clone 11B11; APC), and TNF-α (clone MP6-XT22; Alexa Fluor 700).
Validation	all mAbs used according to manufacturer's instructions and previously published methods; mAbs were validated and titrated for specificity prior to use

Eukaryotic cell lines

Policy information about cell lines and Sex and Gender in Research

Cell line source(s)	HEK293T
Authentication	Commercially purchased from ATCC
Mycoplasma contamination	Negative for mycoplasma
Commonly misidentified lines (See ICLAC register)	N/A

Animals and other research organisms

Policy information about studies involving animals; ARRIVE guidelines recommended for reporting animal research, and Sex and Gender in Research

Laboratory animals	Female BALB/c mice (6-8 weeks old) were used for this study.
Wild animals	N/A
Reporting on sex	Female mice were used in the study. This is standard in the field due to female mice doing better in group housing.
Field-collected samples	N/A
Ethics oversight	All animal studies were conducted in compliance with all relevant local, state and federal regulations and were approved by the Beth Israel Deaconess Medical Center and University of North Carolina at Chapel Hill Institutional Animal Care and Use Committees.

Note that full information on the approval of the study protocol must also be provided in the manuscript.

Flow Cytometry

Plots

Confirm that:

☒ The axis labels state the marker and fluorochrome used (e.g. CD4-FITC).

☒ The axis scales are clearly visible. Include numbers along axes only for bottom left plot of group (a 'group' is an analysis of identical markers).

☒ All plots are contour plots with outliers or pseudocolor plots.

☒ A numerical value for number of cells or percentage (with statistics) is provided.

Methodology

Sample preparation	Lungs were perfused with PBS then extracted from mice and processed using the GentleMACS (Miltenyi Biotec). Lung cells were then passed through 30 μm filter. Splenocytes were pushed through a 70 μm filter followed by a 30 μm filter. Both cell types were then surface stained for antibodies previously listed in antibody section. Cells were then permeabilized using BD Cytofix/CytoPerm (BD: AB_2869008). After permeabilization intracellular cytokine stain was conducted with antibodies described above. Cells were then resuspended in 2% Paraformaldehyde solution and run through flow cytometer.
Instrument	BD-LSRII
Software	FLOW data was collected using BD FACSDiva software. FLOW data was analyzed using FLOWjo v10.
Cell population abundance	No sorting.
Gating strategy	The full gating strategy for representative spleen and lung samples are provided in supplemental fig S1 and S3 respectively.

☒ Tick this box to confirm that a figure exemplifying the gating strategy is provided in the Supplementary Information.
